# Next-generation methods in health technology assessment (HTA): need, rigor, and implementability

**DOI:** 10.1017/S0266462326103900

**Published:** 2026-07-07

**Authors:** Marina Richardson, Federico Augustovski, Melissa Pegg, Cyril Seck, Sitanshu Sekhar Kar, Wim Goettsch

**Affiliations:** 1 Institute for Clinical and Economic Review, USA; 2 Institute for Clinical Effectiveness and Health Policy (IECS), Argentina; 3 University of York, UK; 4 Africa CDC, Ethiopia; 5 Jawaharlal Institute of Postgraduate Medical Education and Research, India; 6 Universiteit Utrecht, Netherlands; 7 Healthcare Institute of the Netherlands, Netherlands

**Keywords:** environmental sustainability, adaptive Health Technology Assessment (HTA), artificial intelligence, real-world evidence (RWE), implementation

## Abstract

The Health Technology Assessment International (HTAi) 2025 annual meeting featured three main plenaries to explore next-generation (NextGen) evidence in health technology assessment (HTA). In this commentary, we capture the discussions of Plenary 2: *NextGen Methods: Hype or Here to Stay?* Each plenary panelist was tasked to convincingly debate the need, rigor, and implementability of one of three emerging method domains in HTA: (1) Environmental Sustainability, (2) Adaptive HTA, and (3) Artificial Intelligence (AI)-enabled Real-World Evidence (RWE). The three panelists convincingly debated that their method would endure beyond initial hype; all three methods were perceived to have a moderate to high level of need, rigor, and implementability by the audience. Key questions from the audience included a request for examples of where HTA reviews have considered environmental sustainability, a challenge for adaptive HTA to embrace other value elements outside of cost-effectiveness, and a question about how the human-in-the-loop principle fits into AI-driven RWE and what this means for HTA agencies that are already stretched for resources. In this commentary, we summarize the presentations, discussions, and audience engagement to provide readers with accessible insight into the debate about which method(s) are anticipated to endure beyond their initial hype.

## Introduction

The Health Technology Assessment International (HTAi) Annual Meeting held in Buenos Aires in June 2025 brought the global HTA community together to explore next-generation (NextGen) evidence, a theme that prompted reflection on how evidence is conceptualized and used in HTA (1). Three plenary sessions carried this theme throughout the conference. Plenary 1 examined whether HTA should move beyond its conventional role of conducting incremental assessments toward becoming a proactive system shaper. 2 Two built on these discussions by exploring the methods needed to respond effectively to emerging innovations, evolving health system needs, and a potential system-shaping role for HTA. Plenary 3 concluded the series with a critical examination of participation and the importance of building trust through transparency, equity, and fair health systems. While the present manuscript focuses on the discussion from Plenary 2, *“NextGen Methods – Hype or Here to Stay,”* the content of the other plenaries is presented by other authors as separate contributions.

Plenary 2 selected and focused on three key method domains that are arguably at their peak of expectations, ones that have a lot of promise but are not yet part of the mainstream. The three key domains of methods explored were as follows: (1) Environmental Sustainability, (2) Adaptive HTA, and (3) Artificial Intelligence (AI)-enabled Real-World Evidence (RWE). Inspired by the work of SUSTAIN-HTA (2), the panelists were asked to convince the audience of the strength of their method domain using a three-part framework of Need, Rigor, and Implementability. Each speaker allocated one slide to each part of the framework to help shape the debate about which method(s) are anticipated to endure beyond their initial hype. Borrowing from the stages outlined in the Gartner Hype Cycle (a model for anticipated stages of innovation adoption)(3), the plenary explored how each method will fare over the next 2–10 years. Triggering questions included: Is the method ready for prime time now? Will it reach its peak of inflated expectations only to meet a trough of disillusionment? Will there be another window of time where the need is even greater? Is it only a matter of time before the method hits a plateau of productivity where it is embedded in HTA processes?

A “challenger” role was featured as part of the discussion with the aim of asking questions to understand what is at the heart of the method that each speaker represented, probe for specific examples of practical applications of their method, and to seek clarity on what might help or hinder adoption by HTA bodies. Audience participation was encouraged, and during each presentation, the audience was asked a rating-scale question (from 1 to 10, with 10 being the highest) on how they viewed the strength of the need, rigor, and implementability of each of the presented methods.

This commentary reports the current state of the art of each of the methods, captures the insights, questions, and debate about which method(s) are anticipated to endure beyond their initial hype. A summary of the Need, Rigor, and Implementability of each method of interest is shown in Table 1.

**Table 1.** Summary of the need, rigor, and implementability of environmental sustainability, adaptive HTA, and AI and RWE in HTA Table 1. long description.

Key highlights			
Dimension	Environmental sustainability	Adaptive HTA	AI and RWE
Need	HTA must evolve to address the rapid emergence of complex healthcare technologies and integrate broader societal and environmental considerations, ensuring decisions support both human and planetary health.	Adaptive HTA complements a full HTA when specific situational triggers are met, including: urgency (e.g., during pandemics), certainty (e.g., when the evidence is available from high-quality studies from a similar context), and budget impact (e.g., in cases with a low budget impact technology, a rapid assessment can guide fiscal policy).	Accelerated regulatory approvals based on immature or non-randomized evidence, together with near-universal adoption of electronic health records, have created a structural requirement for AI-enabled real-world evidence in health technology assessment.
Rigor	Adoption of internationally recognized environmental and methodological standards, such as the GHG Protocol, ISO 14040/44, PAS 2050, and PAS 2090, combined with clinician and stakeholder input, ensures robust, transparent, and globally comparable assessments.	Allows policymakers to act decisively without abandoning scientific grounding. aHTA can synthesize available disparate data to provide working guidance and significantly reduce the analytic burden on HTA bodies, allowing them to allocate resources more efficiently.	AI-enabled real-world evidence can meet established HTA standards when embedded within transparent study designs, validated analytic pipelines, and explicit safeguards addressing bias, data quality, and reproducibility.
Implementability	Practical frameworks, adaptive methods, and collaborative engagement with healthcare professionals, regulators, and decision-makers enable HTA to be actionable, context-sensitive, and feasible across diverse healthcare systems and resource settings.	Integrating aHTA requires a shift in philosophical perspective. aHTA is imperfect, perhaps incomplete, and transient, yet it possesses a distinct value in its utility. By accepting the imperfections of aHTA, we gain the ability to make timely, life-saving decisions that a pursuit of perfection would delay.	Federated data infrastructures and formal regulatory frameworks demonstrate that AI-enabled real-world evidence can be operationalized within existing HTA processes, provided that interoperability, governance, and analytical capacity are in place.

aHTA, adaptive health technology assessment; AI, artificial intelligence; HTA, health technology assessment.

## Methods explored and audience participation

### Method 1: Environmental sustainability

#### Need

The environmental impact of healthcare systems is substantial, contributing approximately 5% of annual greenhouse gas (GHG) emissions across the world (4) and up to 8.5 percent of annual GHG emissions in the United States, exceeding the total emissions of many nations (5). Without intervention, healthcare emissions could reach six gigatons annually by 2050, equivalent to 1.26 billion cars (6). This magnitude underscores the urgent need for healthcare decision-makers to integrate environmental sustainability within evaluative frameworks, to ensure that interventions designed to improve human health do not simultaneously compromise planetary health. The imperative aligns with commitments made by 143 countries at COP28 to transform health systems into low-carbon, climate-resilient, and equitable structures (7).

#### Rigor

Embedding environmental sustainability in HTA demands the same scientific rigor applied to clinical and economic evaluations, including transparent quantification of carbon and resource impacts (8). Moreover, the need to integrate environmental sustainability into HTA is being increasingly recognized as requiring explicit trade-off considerations (9), potentially using weighting, thresholds, or ceiling ratio approaches to balance environmental, clinical, and economic value (10). For example, beyond descriptive assessment, value frameworks could evolve to incorporate weighted environmental costs – either within the financial domain (as a monetized externality) or as an additional outcome dimension, reflecting ecological benefit or harm (11). This expansion would allow cost-effectiveness, cost–benefit, or cost-comparison analyses to more accurately represent the broader societal value of interventions. The inclusion of environmental impact within pricing and reimbursement considerations could, in turn, incentivize sustainable innovation, rewarding low-carbon, resource-efficient technologies and encouraging industry investment in green design and manufacturing practices (12).

#### Implementability

The development of environmental sustainability within HTA requires an interdisciplinary approach integrating clinical, economic, and environmental expertise. The active engagement of healthcare professionals is particularly important to ensure that environmental assessment methodologies align with real-world technology use and clinical workflows, enhancing credibility, relevance, and feasibility (13). Despite growing momentum, challenges persist, including data limitations, inconsistent geographical boundaries, and regional variation in priorities (14). Achieving implementation of environmental sustainability in HTA requires adaptive, context-sensitive approaches, leveraging AI, predictive modeling, and real-world data. By balancing need (the urgency of reducing healthcare’s environmental footprint), rigor (the robust and transparent quantification of sustainability impacts), and “implementability” (the feasibility of applying these methods within real-world clinical and procurement pathways), HTA can evolve into a new generation, helping to deliver technologies that both benefit human and planetary health.

### Method 2: Adaptive HTA

#### Need

In the evolving landscape of global healthcare, decision-makers are constantly balancing the need for rigorous scientific evidence with the exigencies of real-world policy implementation. Traditional HTA provides a robust framework for evaluating the impacts of health technologies in terms of costs and health effects. However, traditional, or full HTA, is often time-consuming and resource-intensive. This creates a critical knowledge gap where decisions must be made before a full assessment can be completed. As noted in recent scoping reviews, “adaptive” or “rapid” HTA serves as a necessary evolution to address these constraints (15). Table 2 highlights some of the benefits and limitations of full and adaptive HTA methods.

**Table 2.** Key differences between full and rapid review adaptive HTA Table 2. long description.

	Full HTA	Adaptive HTA	Benefits of aHTA	Limitations of aHTA
Timeline	12–24 months+	1–6 months	• Speed	• Level of comprehensiveness
Analysis	De novo clinical and economic evaluation (e.g., cost-effectiveness analysis)	Review of HTAs and other published cost-effectiveness evidence	• Requires less resources, staff time, and skills • Leverages high-quality analysis and appraisal capacity from other HTAs	• Lower accuracy • (Un)certainty
Data sourcing	Local studies + primary data collection and systematic literature review	International HTA reports + targeted literature search for economic studies	• Speed of data sourcing • Leverages other HTA agencies’ access to data	• Level of comprehensiveness • Reduced confidence in transferability to local context
Uses	• Adjustments to health benefits packages or essential medicines lists • Adoption of individual technologies • Price negotiations • Reimbursement decisions • Clinical guidelines • Quality standards	Same as HTA	• Greater speed and quantity of decisions informed by aHTA may improve the overall allocative efficiency of the benefits package	• Lower precision of aHTA makes price negotiation less informed • Greater uncertainty of aHTA risks error in adjustment to packages • Not possible to do topics with no preexisting international HTAs

*Note*: This table is taken from the iDSI Guide to Rapid Review of International HTA Reports: An Adaptive HTA Method (33).

aHTA, adaptive health technology assessment; AI, artificial intelligence; HTA, health technology assessment.

Adaptive HTA (aHTA) is not meant as a shortcut, but rather a strategic tool. It allows for evidence-based decision-making even – or especially – when time and resources are more limited.

##### Triggers for aHTA

Adaptive HTA does not replace full HTA but rather complements it when specific situational triggers are met. Three primary drivers for the use of an adaptive approach are provided below:

1. Urgency: In situations such as pandemics or rapid policy shifts, the timeline for a full HTA, which can take months or years, is untenable. The urgency of the situation demands a faster, more swift response.
2. Certainty: The level of evidence required or currently available impacts the choice of assessment. When decision-making cannot wait for perfect data, aHTA allows for an assessment based on the currently available level of certainty.
3. Budget Impact: High-stakes financial decisions often require robust evidence and analysis to justify spending or cost-cutting. A low budget impact for a technology can trigger the need for a rapid assessment to guide policy.

These triggers align with international frameworks that define aHTA as a structured approach to adjusting analysis based on available time, data, and capacity (15).

#### Rigor

##### Balancing efficiency and rigor: strengths and weaknesses

The utility of aHTA is best understood through a critical analysis of its operational strengths and limitations. The primary advantage of aHTA is its ability to facilitate quick, evidence-based decisions. Streamlining the assessment process allows policymakers to act decisively without abandoning scientific grounding. Furthermore, in novel situations where comprehensive trials are lacking, aHTA may use available disparate data to provide working guidance. Operationally, this approach may significantly reduce the analytic burden on HTA bodies, allowing them to allocate scarce resources more efficiently. Importantly, engaging in aHTA often creates an impetus for future full HTA work. It can serve as a preliminary scoping exercise that highlights gaps in knowledge, justifying and guiding subsequent, more detailed investigations (16).

Despite its practical benefits, aHTA is not without challenges. A relevant example is the transferability of data from other settings. Rapid assessments often rely on international data or proxy datasets, which may not be perfectly applicable to the local context (17). Additionally, the simplified nature of aHTA may not build wide-ranging capacity for all types of HTA, potentially limiting the skill development required for more complex economic modeling. There is also a lack of a standardized approach for aHTA. Unlike the protocols of full HTA, adaptive methods vary significantly, which can lead to inconsistencies in quality and reporting. Finally, this method is particularly challenging for complex interventions, where multiple interacting components require the deep, granular analysis that only a full HTA can provide (18).

#### Implementability: The philosophy of imperfection

To truly integrate aHTA into our decision-making frameworks, a shift in philosophical perspective is required. This can be conceptualized through the Japanese aesthetic of “Wabi-sabi.” Wabi-sabi is a worldview that focuses on finding beauty in imperfection and accepting the natural cycle of growth and decay. It teaches that “nothing lasts and nothing is perfect” (19).

In the context of health technology assessment, full HTA represents the pursuit of perfection complete data, exhaustive analysis, and minimal uncertainty. However, the real world is imperfect. aHTA represents the *wabi-sabi* of healthcare policy: it is imperfect, perhaps incomplete, and transient, yet it possesses a distinct value and “beauty” in its utility. By accepting the imperfections of aHTA (such as reduced data certainty or transferability issues), we gain the ability to make timely, life-saving decisions that a pursuit of perfection would delay.

Adaptive HTA is not a compromise in quality, but a strategic adaptation to reality. It acts as a bridge between the rigorous demands of science and the urgent needs of policy. While it presents challenges regarding standardization and data transferability, its ability to reduce analytic burden and provide rapid evidence makes it indispensable.

Ultimately, aHTA is another tool in our toolbox. It allows for flexibility, ensuring that the HTA community can serve the healthcare ecosystem effectively, even when conditions are far from perfect. Future efforts should focus on standardizing these adaptive methods to mitigate their weaknesses while maximizing their inherent agility, and each country’s HTA ecosystem should decide how to use this tool in health policy decision-making.

### Method 3: Artificial intelligence-enabled RWE

#### Need

AI and RWE are increasingly central to current HTA practices. Across health systems, assessors face accelerated innovation cycles, particularly in oncology, advanced biologics, and digital health technologies, where evidence at the time of market entry is frequently incomplete. Systematic reviews show that major HTA agencies already rely on real-world data (RWD) to complement randomized controlled trials (RCTs), notably to assess external validity, long-term outcomes, utilization patterns, and real-world effectiveness (20;21).

This shift coincides with the widespread digitization of health systems. The OECD reports that 90 percent of hospitals in surveyed high-income countries have implemented electronic health records (EHRs), generating large volumes of routinely collected data suitable for secondary analysis (22). Advances in machine learning and natural language processing (NLP) may now make it possible to curate and analyze these data at scale. As a result, AI-enabled RWE may be shifting from a supplementary role toward a structural component of HTA.

##### Structural drivers of demand for AI-enabled RWE

The growing prevalence of regulatory approvals based on non-randomized or immature evidence has intensified reliance on RWE. An analysis of Food and Drug Administration (FDA) oncology approvals between 2002 and 2021 found that 31% were supported by non-randomized studies, including single-arm trials (23). Such evidence is insufficient for comparative value assessment, requiring HTA bodies to generate complementary real-world analyses to characterize effectiveness and uncertainty (20).

Concurrently, the scale and complexity of available real-world data (RWD) have expanded beyond the capacity of conventional analytic workflows. In Europe, the European Medicines Agency’s (EMA) DARWIN initiative exemplifies this transformation. By 2024, DARWIN comprised more than 30 data partners and provided federated access to over 100 million patient records harmonized to the Observational Medical Outcomes Partnership (OMOP) common data model (24). These infrastructures enable large-scale observational analyses while preserving data sovereignty. In such environments, AI-based methods are increasingly necessary to ensure timely, reproducible, and auditable evidence generation.

Regulatory frameworks have evolved in parallel. The FDA’s RWE Program defines expectations for data relevance, reliability, and study design when RWE informs regulatory decisions (25). In Europe, the EMA has embedded RWE within its regulatory strategy through DARWIN and methodological guidance on big data and data quality (26). The National Institute for Health and Care Excellence (NICE) has formalized expectations through its RWE Framework, specifying criteria for data suitability, causal inference, and transparency in HTA submissions (27). As regulatory standards converge, HTA bodies face growing pressure to align their evidentiary approaches.

#### Rigor

The methodological basis for using RWE in HTA is well established. Systematic reviews indicate that HTA agencies routinely rely on observational studies to inform epidemiology, long-term outcomes, resource use, and external validity, provided that transparent protocols and appropriate causal inference methods are applied (20;21). Techniques such as propensity score adjustment and target trial emulation are now standard in high-quality RWE.

AI methods act primarily as analytic enablers applied to these established designs. NLP has demonstrated strong performance in extracting clinically relevant variables from unstructured EHR text, with studies reporting precision ranging from 0.75 to 1.0 and recall from 0.71 to 1.0 across validated oncology applications (28). Similarly, work by the Observational Health Data Sciences and Informatics community shows that automated phenotyping within harmonized data models improves reproducibility and scalability across databases (29). When rigorously validated, these tools can strengthen the evidentiary basis of HTA by reducing manual abstraction error and enhancing consistency.

Nonetheless, risks related to bias, representativeness, and interpretability are well documented. The World Health Organization (WHO) warns that AI systems trained on incomplete or unrepresentative datasets may amplify existing biases unless explicit safeguards are implemented (30). The EMA’s Big Data Steering Group similarly highlights heterogeneity in data completeness, coding practices, and population coverage across European RWD sources, underscoring the need for systematic data quality assessment (26). For HTA, AI-generated evidence must, therefore, be evaluated not only for statistical performance but also for transparency, fairness, and transportability.

Governance frameworks provide mechanisms to address these risks. NICE’s RWE Framework requires protocol registration, assessment of data fitness for purpose, and explicit causal reasoning (27). EMA guidance on registry-based studies specifies expectations for data governance, curation, and reproducibility (31). WHO guidance emphasizes auditability and human oversight as core principles for trustworthy AI in health (30). These safeguards are directly applicable to AI-enabled RWE without altering foundational HTA principles.

#### Implementability

Evidence from early adopters indicates that AI-enabled RWE can be operationalized within existing HTA processes. DARWIN EU demonstrates a federated model capable of supporting large-scale observational analyses relevant to regulatory and HTA questions without centralizing patient-level data (24). NICE has increasingly incorporated RWE into re-assessment, managed access agreements, and horizon scanning, illustrating practical integration within HTA workflows (27).

Successful implementation depends on several institutional conditions. Interoperable data infrastructures based on common data models facilitate reproducible analyses across jurisdictions (29). Clear governance arrangements, including documentation of data provenance and analytic pipelines, are essential for accountability (26). In addition, HTA organizations require analytical capacity to critically appraise AI methods and their underlying assumptions (32). Absent these conditions, AI risks increasing opacity rather than improving decision quality.

## Summary of discussions and audience rankings – which method(s) are here to stay?

During Plenary 2 of HTAi 2025, and as described above, we heard about the implications of climate change and the role that HTA can play, the need for efficiency in evidence reviews and cost-effectiveness modeling, and the opportunities for AI and RWE to support a health-system-shaping role. We also heard from the audience and our “challenger” with probing and thoughtful questions to dig deep into why each method is needed and is set to endure beyond its initial hype. For environmental sustainability, the audience asked the speaker to share examples of where HTA reviews have considered environmental sustainability, and if environmental considerations are needed for all assessments. For adaptive HTA, the audience challenged the method to embrace other value elements outside of cost-effectiveness. For AI and RWE, the question was how the human-in-the-loop principle fits into AI-driven RWE and what this means for HTA agencies that are already stretched for resources. The top three questions asked and responses from the panel speakers are summarized in the Supplementary Material.

**Figure 1.**
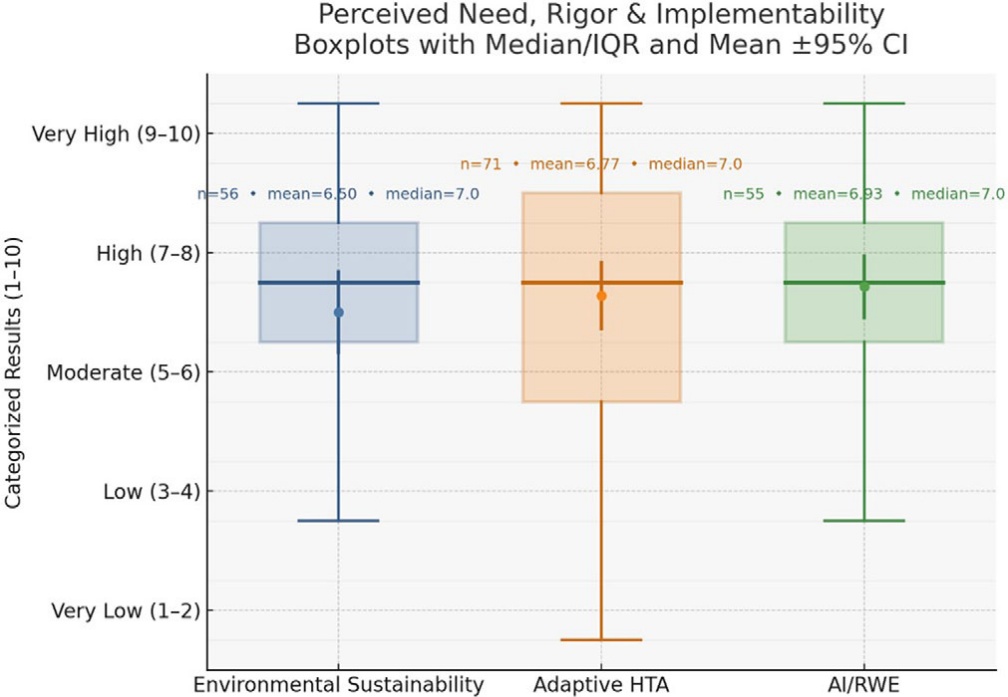
Audience voting results for the perceived need, rigor, and implementability of each method domain (environmental sustainability, adaptive HTA, and AI-enabled RWE). AI, artificial intelligence; CI, confidence interval; HTA, health technology assessment; IQR, interquartile range; RWE, real world evidence. Figure 1. long description.

Figure 1 illustrates the voting results from the audience, which demonstrate that the panelists convincingly debated that their methods would endure beyond initial hype. On a scale of one to ten, all three methods were perceived to have a moderate to high level of need, rigor, and implementability (mean scores ranging from 6.5 to 6.9), with median scores (all scores were 7.0) representing a high level of perceived need, rigor, and implementability. It is unclear if need, rigor, or implementability drove the voting across methods. However, it was clear from the speakers’ passion, the audience participation, and the energy of the conference that there is a call to action for the HTA community to continue to work on these methods to ensure they are responding to health system needs, have the scientific rigor to ensure trust, and the importance of engaging with decision-maker needs to have methods that are implementable.

Importantly, through probing questions, the audience captured the importance of considering the interplay between disciplines: *“How does the use of AI in HTA reconcile with environmental sustainability since we know that the use of AI is associated with significant carbon footprint?”* We are a global community with global opportunities and challenges, and it is the culmination of disciplines and their interplay that brings life to HTA. We encourage readers, researchers, and the global community to work together to continue to push the boundaries of these methods to support an equitable, efficient, and high-quality health system.

## Supporting information

10.1017/S0266462326103900.sm001 Richardson et al. supplementary material Richardson et al. supplementary material

## Supplementary material

The supplementary material for this article can be found at http://doi.org/10.1017/S0266462326103900.

## Long descriptions

### Table 1. Long description

The table is organized into four columns and four rows. The first column lists the dimensions of evaluation, while the subsequent columns cover three specific areas: Environmental sustainability, Adaptive H T A, and A I and R W E.

* Row 1: Dimension: Need.

- Environmental sustainability: H T A must evolve to address complex healthcare technologies and integrate societal and environmental considerations for human and planetary health.

- Adaptive H T A: Complements full H T A during urgency (pandemics), certainty (high-quality evidence), or low budget impact.

- A I and R W E: Accelerated approvals and electronic health records create a structural requirement for A I-enabled real-world evidence.

* Row 2: Dimension: Rigor.

- Environmental sustainability: Uses international standards like G H G Protocol, I S O 14040/44, P A S 2050, and P A S 2090 with stakeholder input.

- Adaptive H T A: Synthesizes disparate data to provide guidance and reduce analytic burden on H T A bodies.

- A I and R W E: Meets standards when embedded in transparent designs, validated pipelines, and safeguards against bias.

* Row 3: Dimension: Implementability.

- Environmental sustainability: Practical frameworks and collaborative engagement enable actionable, context-sensitive H T A.

- Adaptive H T A: Requires a shift to accept imperfect, transient data to make timely, life-saving decisions.

- A I and R W E: Federated data infrastructures and regulatory frameworks show operational feasibility through interoperability and governance.

Navigate back to Table 1..

### Table 2. Long description

The table is organized into five columns: Category, Full H T A, Adaptive H T A, Benefits of a H T A, and Limitations of a H T A.

* Timeline: Full H T A takes 12 to 24 months or more, while Adaptive H T A takes 1 to 6 months. The benefit is speed, while the limitation is the level of comprehensiveness.

* Analysis: Full H T A involves de novo clinical and economic evaluation like cost-effectiveness analysis. Adaptive H T A reviews existing H T A s and published evidence. Benefits include requiring fewer resources and leveraging high-quality appraisals from others. Limitations include lower accuracy and uncertainty.

* Data sourcing: Full H T A uses local studies, primary data, and systematic literature reviews. Adaptive H T A uses international H T A reports and targeted searches. Benefits include speed and access to other agencies’ data. Limitations include reduced confidence in local context transferability.

* Uses: Both types are used for health benefit package adjustments, technology adoption, price negotiations, reimbursement, clinical guidelines, and quality standards. Benefits of Adaptive H T A include improved allocative efficiency through faster decision-making. Limitations include lower precision for price negotiations, risk of error due to uncertainty, and inability to assess topics without preexisting international reports.

Navigate back to Table 2..

### Figure 1. Long description

The Y-axis is labeled Categorized Results (1-10) with five levels: Very Low (1-2), Low (3-4), Moderate (5-6), High (7-8), and Very High (9-10). The X-axis lists three domains from left to right:

* Environmental Sustainability (Blue): n = 56, mean = 6.50, median = 7.0. The box spans from approximately 5.5 to 8.5. A central dot represents the mean with a vertical error bar for the 95% C I.

* Adaptive H T A (Orange): n = 71, mean = 6.77, median = 7.0. The box spans from approximately 5.5 to 8.5. The lower whisker extends down to the Very Low range.

* A I / R W E (Green): n = 55, mean = 6.93, median = 7.0. The box spans from approximately 6.5 to 8.5.

All three domains show a median score of 7.0, falling in the High category, with A I / R W E showing the highest mean score and the most concentrated distribution in the upper quartiles.

Navigate back to Figure 1..
