# Supplementary material for: Next-generation methods in health technology assessment (HTA): need, rigor, and implementability
Source: Int J Technol Assess Health Care. 2026 Jul 7;42(1):e58. doi: 10.1017/S0266462326103900 (PMC13434855; doi:10.1017/S0266462326103900)
Supplement: Supplementary file 1 [file S0266462326103900sup001.docx]

### Supplemental Material

| **Questions from the Audience** |  |
| --- | --- |
| **General Question** | **Upvotes** |
| Many treatments that are currently reimbursed do not meet today's HTA standards, should we not focus more strongly on this than on what's new in HTA? | 0 |
| **Environmental Sustainability** |  |
| Do we have examples where environmental sustainability was included in the HTA? | 7 |
| Wouldn't be mandatory the environmental assessment for every product? Not just medicine or medical procedures? | 1 |
| What is the minimum environmental aspects you suggest to be included in HTA | 7 |
| Surely overuse of medicines, safe disposal of unused medicines, medicine reviews that sense-check Polypharmacy should be the first step? | 2 |
| Is environmental assessment a first world problem? | 2 |
| If we are willing to pay for environmental benefits derived from implementing some health technology, how [do] we adapt methods for capturing opportunity costs? | 2 |
| There are many initiatives for Sustainability-HTA eg SMI and HTAi with multi-stakeholders. Is there any international effort to connect all those initiatives? | 0 |
| If the majority of HTA activity is assessing drug A vs B, there may not be any significant differences in environmental impacts between options? | 0 |
| First step might be to understand whether the impact is significant compared to other industries or sectors to determine whether it's worth including in HTA? | 1 |
| What about all the medicines already on the market? Unless this is assessed retrospectively, we will only be addressing a tiny part of the issue. | 1 |
| Wrong assumptions inevitably lead to wrong conclusions. That’s all about environmental HTA | 0 |
| In developing countries, should we address other social determinants of health first, like nutrition issues and poor living conditions that affect health? | 1 |
| It is hard to envisage a scenario where we do not recommend an otherwise effective and cost-effective technology for an unmet need due to environmental factors. | 0 |
| Is it possible to reach a balance between sustainability vs economic growth, especially in the context of LMIC? | 1 |
| **Adaptive HTA** |  |
| According to your ppt Limitations of aHTA seem more important to its advantages, trying to make it simple sometimes is worse, why this wouldn’t be the case? | 2 |
| Can AI be used in HTA to improve speed and capabilities in countries with limited resources? | 1 |
| Why only cost-effectiveness mentioned in Adaptive HTA and not broader approaches eg multi-criteria, deliberative HTA? | 3 |
| And how is Adaptive HTA in India aligned with the Health Prioritization? | 2 |
| Should adaptive HTA not also embrace other value, outside of cost effectiveness? | 2 |
| Would the aHTA really support decision making in local context if you piggy back on different systemic models? | 0 |
| We could argue that industry does aHTA since we already adapt economic models for many countries. Why not ask manufacturers to submit? | 2 |
| Why is only cost-effectiveness (with limitations) listed for Adaptive HTA and not broader approaches eg multi-criteria, deliberative HTA? | 1 |
| Is it possible to have de novo HTA but be more adaptive in the context of timeline? | 1 |
| Are you adapting the CUA or just taking the results? | 2 |
| Slides mentioned aHTA considers HTA reports from other countries, so does this restrict aHTA to later launch countries? | 0 |
| INAHTA already developed a distinctive toolkit for adapting HTA. Is this used? | 3 |
| For stakeholders we need to consider caregivers too | 0 |
| **AI and RWE** |  |
| Why do you group AI and RWE together? | 3 |
| Which countries in Africa? | 0 |
| How does the use of AI in HTA reconciles with environmental sustainability since we know that the use of AI is associated with significant carbon footprint? | 7 |
| Currently, bias in RWD is an issue, and RCTs provides reliable evidence. AI will help to reduce bias and incompleteness or other data quality issues of RWD? | 2 |
| Where would the human-in-the-loop principle fit into AI-driven RWE? Would this not then make this too labour intensive for already stretched agencies? | 0 |
| How reliable is generative AI to be used in HTA? | 2 |
| The FDA has just laid off 2,000 employees and started using AI in drug approval. Is there really no space for replacement in the HTA? | 3 |
| Can AI help aide sustainability of HTA. Notting there is already too much work for most HTA agencies to assess. | 2 |

**Responses to the top three Questions from the Audience for Each Method Domain**

Method 1: Environmental Sustainability

**Question 1 (7 votes): Do we have examples where environmental sustainability was included in HTA?**

While environmental sustainability has not yet been included in an HTA submission, several HTA agencies provide illustrative examples. In England, NICE has established an HTA Innovation Laboratory to develop and test methodologies for comparing the environmental impacts of health technologies (1), embedding sustainability considerations into topic prioritization, and aligning with broader NHS net-zero ambitions (2). Similarly, Canada’s CDA-AMC has published reports on reducing the environmental impact of clinical care, with strategic plans emphasizing methodological adaptations to incorporate environmental perspectives (3). INESSS in Quebec has developed a strategy to systematically determine when and how environmental considerations are integrated into knowledge products, supported by existing institute projects and its 2024–2028 Strategic Plan (4). The Zorginstituut Netherlands (ZIN) aligns with European legislation to reduce the environmental footprint of medicines, reflecting a commitment to sustainable healthcare innovation (5).

**Question 2 (7 votes): What are the minimum environmental aspects you suggest being included in HTA?**

To integrate environmental sustainability into HTA, assessments could address key life cycle aspects that shape environmental impact. Carbon footprint and GHG emissions are foundational, encompassing direct and indirect emissions from production, distribution, use, and end-of-life stages, guided by standards such as the GHG Protocol (6), ISO 14040/44 (7), PAS 2050 (8) and the forthcoming PAS 2090 (9). Resource use and material consumption could also be evaluated, including critical materials (plastics, metals, electronics) and energy inputs across the life cycle.

Circularity is proposed to be a core principle of sustainable healthcare decision making, guided by the 9Rs framework: refuse unnecessary products, rethink design and use, reduce material and energy demands, reuse, repair, refurbish, remanufacture, and repurpose components, recognizing that recycling should be a last resort (Figure A (The 9Rs framework). This hierarchy promotes sustainable design, product longevity, and material efficiency while minimizing life cycle impacts (10).

**Supplementary Figure A**. The 9Rs framework guide sustainable design, product longevity, and material efficiency while minimizing life cycle impacts.

Evaluating supply chain impacts, including transport, logistics, and production variability, is also critical as they contribute substantially to a product’s total environmental footprint (10). Context-specific effects such as local pollution, water use and ecosystem disruption could be considered to reflect geographically relevant impacts (11). Finally, HTA could align with planetary health principles, linking assessments to frameworks such as the UN Sustainable Development Goals and regional net-zero and circular economy strategies. Collectively, these minimum elements provide a rigorous yet feasible foundation for embedding environmental sustainability into HTA, ensuring frameworks remain globally relevant and actionable (10).

**Question 3 (2 votes): Is environmental assessment a higher resource setting issue?**

Environmental assessment in HTA is not solely a problem in higher resource settings, although resource availability and data infrastructure can influence feasibility. While higher resources settings may have greater access to life cycle data, standardized methodologies, and funding to implement comprehensive environmental assessments, the environmental impacts of healthcare technologies are global. For example, global supply chains, carbon emissions, and resource consumption associated with pharmaceuticals, medical devices, and digital health tools affect both higher and lower resource settings.

Emerging HTA frameworks, such as approaches and methods published by the Health Technology Assessment International (HTAi) Environmental Sustainability in Health Technology Assessment (ESHTA) Working Group (12), International Network of Agencies for Health Technology Assessment (INAHTA), SUSTAIN-HTA (13), and EDiHTA (14), demonstrate that environmental assessment can be adapted for diverse contexts and jurisdictions worldwide, by prioritizing screening-level approaches, life cycle thinking, and practical sustainability indicators. Moreover, environmental considerations intersect with equity and access, as populations in lower resource settings may be disproportionately affected by environmental degradation, climate change, and resource scarcity (15).

By integrating scalable, adaptable methods—such as carbon footprint estimation, resource use assessment, and circularity principles—HTA can support sustainable healthcare decision-making globally. Environmental assessment is therefore a shared responsibility, essential for protecting both human health and planetary health across all countries, not just higher resources contexts.

Method 2: Adaptive HTA

**Question 1 (3 votes): Why is it only cost-effectiveness mentioned in Adaptive HTA and not broader approaches e.g. multi-criteria, deliberative HTA?**

Adaptive HTA (aHTA) prioritizes cost-effectiveness primarily for speed and standardization. When decisions must be made rapidly with evolving data, cost-effectiveness provides a quantitative "litmus test" that is easier to scale and compare than complex qualitative frameworks.

Broad approaches like Multi-Criteria Decision Analysis (MCDA) require high-quality data across multiple domains (equity, social impact, feasibility). Adaptive HTA, by definition, deals with uncertain or evolving data, making it difficult to populate complex multi-criteria matrices accurately in the early stages. Hence, broad approaches like MCDA and deliberative processes are often omitted from the initial "adaptive" phase because they require significant time for stakeholder consensus and high-quality data across multiple domains - both of which are scarce in urgent settings. Consequently, aHTA focuses on the technical evidence core, while broader social and ethical deliberations typically occur later during the final appraisal stage.

**Question 2 (3 votes): INAHTA has already developed a distinctive toolkit for adapting HTA. Is this used?**

INAHTA Adaptation Toolkit (developed in collaboration with EUnetHTA) is a recognized and utilized resource, though its application varies significantly depending on the maturity and resources of the HTA agency. The toolkit is a collection of resources that helps the user assess whether data and information in existing HTA reports should and could be adapted for their own setting. These resources are in the form of checklists of questions on relevance, reliability, and transferability of data and information and links to useful Web sites. The domains covered by the toolkit are technology use, safety, effectiveness, economic evaluation, and organisational aspects.

In practice, the toolkit is not a "plug-and-play" software but rather a methodological framework used to ensure that evidence generated in one country (like the UK or Thailand) can be safely and accurately used in another (like India). Before adopting any toolkit, the contextual factors should be kept in mind.

**Question 3 (Votes 2): We could argue that industry does aHTA since we already adapt economic models for many countries. Why not ask manufacturers to submit?**

While the pharmaceutical industry frequently employs "Global Value Dossiers" to adapt economic models for various markets, public health bodies cannot simply outsource Adaptive HTA to manufacturers due to critical differences in intent and transparency. Industry models are inherently susceptible to "optimism bias," often utilizing selective clinical trial data or favorable long-term assumptions that result in lower Incremental Cost-Effectiveness Ratios (ICERs). Furthermore, manufacturers often compare their products against high-cost competitors rather than the local standard of care, which is essential for national budget decisions.

The "black box" nature of proprietary models also conflicts with the public accountability required for government spending; aHTA necessitates reproducible, open-source modelling that independent bodies can audit and update as real-world evidence evolves. Instead of full outsourcing, many mature systems utilize a hybrid approach: manufacturers submit the technical evidence and base models, while independent academic hubs apply a "trust but verify" filter. This allows agencies to use industry resources to gain speed while maintaining the neutrality needed to ensure the technology truly serves the public interest and addresses local health priorities.

**Method 3: Artificial Intelligence-enabled Real World Evidence**

**Question 1 (3 votes) Why do you group AI and RWE together?**

AI and RWE are grouped together because they address complementary constraints within contemporary HTA. RWE expands the evidentiary base beyond randomized controlled trials by leveraging routinely collected data, but its scale, heterogeneity, and longitudinal complexity often exceed the capacity of traditional analytic methods. AI does not constitute a new evidence source, rather, it functions as an enabling analytic layer that allows RWE to be curated, harmonized, and analyzed with sufficient speed and reproducibility. Grouping AI and RWE reflects a functional relationship: RWE supplies breadth and contextual relevance, while AI provides the methodological capacity required to transform these data into decision-grade evidence.

**Question 2 (7 votes): How does the use of AI in HTA reconcile with environmental sustainability since we know that the use of AI is associated with significant carbon footprint?**

Concerns regarding the environmental footprint of AI are legitimate, particularly for energy-intensive model training and large-scale computational infrastructures. However, most applications of AI in HTA differ fundamentally from frontier model development. HTA-relevant use cases typically rely on comparatively lightweight machine-learning models, rule-based NLP, or federated analytics applied to existing datasets, rather than repeated training of large generative models. Consequently, their marginal carbon footprint is substantially lower.

More importantly, sustainability should be assessed at the system level rather than through isolated computational metrics. AI-enabled HTA can reduce duplicative analyses, accelerate evidence updates, and support earlier disinvestment from low-value technologies, generating downstream efficiency gains within health systems. Moreover, federated data architectures increasingly used for real-world evidence limit data transfer and centralized storage, further reducing energy demand. Under appropriate governance, the environmental costs of AI in HTA are likely to remain modest relative to the system-wide efficiencies it enables.

**Question 3 (3 votes): The FDA has just laid off 2,000 employees and started using AI in drug approval. Is there really no space for replacement in the HTA?**

The introduction of AI into regulatory and HTA processes should not be interpreted as a straightforward substitution of human expertise. While recent staffing reductions at the FDA coincide with increased use of AI tools, this reflects organizational restructuring rather than evidence that AI can replace core evaluative functions. HTA relies on judgement-intensive activities, including problem framing, comparator selection, interpretation of uncertainty, ethical reasoning, and deliberation over value, all of which remain irreducibly human.

AI systems are well suited to automating bounded, repetitive, or data-intensive tasks such as literature screening, data extraction, signal detection, or scenario testing. In this sense, AI enables task substitution at the margin, not role replacement. Its principal contribution lies in augmenting analytical capacity, allowing assessors to focus on higher-order interpretation and decision making rather than mechanical processing.

From an institutional perspective, the relevant question is not whether AI replaces HTA professionals, but how it reshapes skill requirements. Greater emphasis will be placed on methodological oversight, critical appraisal of algorithms, and governance of automated evidence pipelines. Rather than shrinking the role of HTA, AI is more likely to increase the demand for analytically literate assessors capable of integrating complex evidence into accountable public decisions.

**References**

1. National Institute of Health and Care Excellence. HTA Lab projects. Available from: <https://www.nice.org.uk/what-nice-does/our-research-work/hta-lab/hta-lab-projects>
2. NHS England. Delivering a net zero National Health Service. Available from: <https://www.england.nhs.uk/greenernhs/a-net-zero-nhs/>
3. Parker G, Miller FA. Environmental sustainability in health technology assessment: an analysis of the activities of Canada’s Drug Agency and the England’s NICE. International journal of technology assessment in health care. 2025;41(1):e53.
4. INESSS. Environmental impacts on health and social services. Available from: <https://www.inesss.qc.ca/en/metho-et-veille/demarche/nos-publications-de-veille-scientifique/impacts-environnementaux-en-sante-et-en-services-sociaux.html?utm_source=chatgpt.com>
5. National Institute for Public Health and the Environment. Improve method can provide greater insight into the environmental impact of the Dutch healthcare system. 2025. Available from: <https://www.rivm.nl/en/news/improved-method-can-provide-greater-insight-into-environmental-impact-of-dutch-healthcare?utm_source=chatgpt.com>
6. Greenhouse Gas Protocol. Standards and guidance. Available from: <https://ghgprotocol.org/standards-guidance>
7. International Organization for Standardization. Environmental management - life cycle assessment - principles and framework (ISO 14040:2006). 2006.
8. BSI Group. Assessment of life cycle greenhouse gas emissions from horticultural products (PAS2050-1:2012). 2012.
9. BSI Group. Pharmaceutical products - Product category rules for environmental lifecycle assessments - specification (PAS 2090). Available from: <https://standardsdevelopment.bsigroup.com/projects/9025-12045>
10. Rizan C, Bhutta MF. Reducing the environmental impact of medical devices adopted for use in the NHS. Brighton and Sussex Medical School and University of Sussex; 2024.
11. Williams JTW, Bell KJL, Morton RL, Dieng M. Methods to Include Environmental Impacts in Health Economic Evaluations and Health Technology Assessments: A Scoping Review. Value in Health. 2024;27(6):794-804.
12. Holtorf A-P, Pegg M, Mueller D, McMeekin N. Toward including environmental sustainability in Health Technology Assessment. International journal of technology assessment in health care. 2025;41(1):e70.
13. SUSTAIN-HTA. Homepage. Available from: <https://sustain-hta.org/>
14. EDiHTA. Homepage. Available from: <https://edihta-project.eu/>
15. World Health Organization. Strengthening health resilience to climate change. 2015. Available from: <https://www.who.int/docs/default-source/climate-change/technical-briefing---health-resilience-to-climate-change.pdf>
